# The present and future burden of previously treated advanced non-small cell lung cancer (NSCLC) by histology and line of therapy in France, Germany, Italy, and Spain: model-based predictions

**DOI:** 10.1186/s12963-018-0174-4

**Published:** 2018-11-26

**Authors:** David Campbell, Ken O’Day, Nadine Hertel, John R. Penrod, Melinda Manley Daumont, Michael Lees

**Affiliations:** 1 Xcenda LLC, Palm Harbor, Florida, USA; 2 Worldwide Health Economics and Outcomes Research, Bristol-Myers Squibb Pharmaceuticals Ltd, Uxbridge, UK; 3 Worldwide Health Economics and Outcomes Research, Bristol-Myers Squibb Pharmaceuticals Ltd, Princeton, NJ USA; 4 Worldwide Health Economics and Outcomes Research, Bristol-Myers Squibb Pharmaceuticals Ltd, Rueil-Malmaison, France

**Keywords:** Non-small cell lung cancer, Predictive model, Patient forecast, Patient number estimates, Treatment patterns, Line of therapy, Europe

## Abstract

**Background:**

The burden of advanced non-small cell lung cancer (NSCLC) is not well understood, and the number of patients likely to receive treatment in Europe has not been quantified. The aim of this study was to forecast the annual number of patients with squamous and non-squamous advanced NSCLC likely to receive second and third lines of therapy (LOT) from 2016 to 2020 in France, Germany, Italy, and Spain.

**Methods:**

A patient count model (PCM) was developed in Microsoft Excel to estimate the number of patients with refractory advanced NSCLC eligible to receive systemic treatment. Using historical population-based cancer registry data, segmented linear regression (“Joinpoint”) was used to forecast age- and sex-stratified lung cancer incidence rates in each country through 2020. Yearly incident case count totals by country were apportioned according to NSCLC histology and stage at diagnosis. Country-specific treatment rates came from a recent medical chart review study, and early- to advanced-stage disease progression rates were estimated over a 10-year interval. A probabilistic sensitivity analysis (PSA) was performed to estimate variability in the patient counts.

**Results:**

The combined number of squamous and non-squamous advanced NSCLC patients estimated to receive second and third LOT, respectively, in 2016 were France = 11,600 and 3500; Germany = 15,100 and 4900; Italy = 13,500 and 2500; Spain = 9400 and 2100. The forecasted numbers of patients receiving second and third LOT, respectively, in 2020 were France = 13,900 and 4200; Germany = 16,200 and 5200; Italy = 15,100 and 2600; Spain = 11,000 and 2500.

**Conclusions:**

Driven by growth in the incidence of NSCLC among women, the model forecasts an overall increase in the number of patients with advanced-stage squamous and non-squamous NSCLC likely to receive systemic treatment in the year 2020. The results highlight the significant burden of refractory advanced NSCLC and the need for more robust surveillance data to accurately quantify the burden of disease.

**Electronic supplementary material:**

The online version of this article (10.1186/s12963-018-0174-4) contains supplementary material, which is available to authorized users.

## Background

Lung cancer is the leading cause of cancer-related death in Europe and throughout the world [1]. The World Health Organization (WHO) reports that each year there are more than 400,000 new cases of lung cancer in Europe and 1.8 million cases worldwide [2]. Lung cancer is commonly morphologically classified into small cell lung cancer (SCLC) and non-small cell lung cancer (NSCLC), with NSCLC accounting for approximately 85% of all lung cancer cases [3, 4]. NSCLC can be further subclassified by tumor histology into squamous and non-squamous disease, the latter including both adenocarcinoma and large cell carcinoma [5]. NSCLC clinical treatment pathways and outcomes vary according to the stage at diagnosis, morphology, tumor histology, mutation status, and performance status of the patient [5, 6]. Patients with early-stage localized NSCLC may successfully undergo surgery. However, due to difficulties in diagnosing lung cancer, since symptoms may not appear until the disease has reached an advanced stage, the majority of patients are diagnosed at an advanced stage and are not candidates for surgery [5]. The prognosis for patients with advanced disease is poor, as the five-year survival rate for stage IV NSCLC is only 4% [5].

Due to the significant morbidity and mortality associated with NSCLC, it is important for health authorities to have a strong understanding of the disease epidemiology. However, published estimates of the projected number of incident cases of any type of cancer are limited. Furthermore, observed counts of prevalent patients in various stages of treatment within a defined geographic area have not been published and are not readily available in population-based cancer registries [1]. Dynamic patient count models (PCMs) using regional and national historical incidence rate data to project future incidence patterns by age, sex, and line of therapy (LOT) have been successful at addressing this data gap, with close concordance to GLOBOCAN registry findings [7]. The aim of this study was to forecast the annual number of patients with refractory squamous or non-squamous NSCLC likely to receive second and third LOT from 2016 to 2020 in France, Germany, Italy, and Spain in order to better quantify the burden of disease.

## Methods

### Model design

**Fig. 1:**
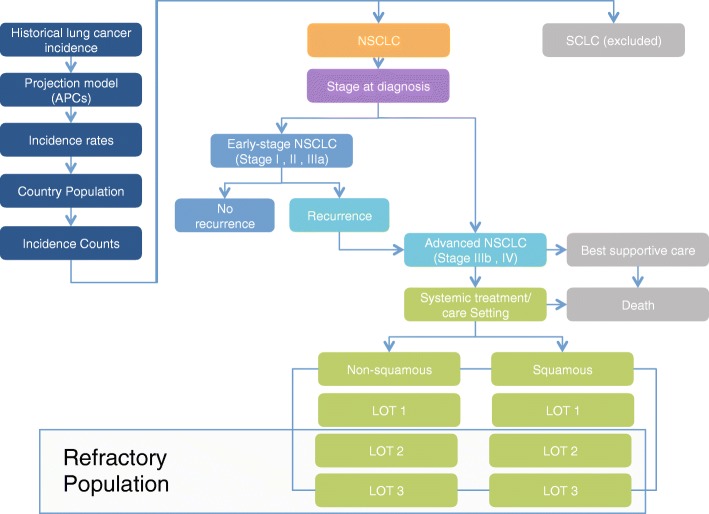
Refractory NSCLC patient count model structure. Key: APC – annual percent change; LOT – line of therapy; NSCLC – non-small cell lung cancer; SCLC – small cell lung cancer

An Excel-based PCM was developed to forecast the number of previously treated advanced NSCLC patients in France, Germany, Italy, and Spain from 2016 to 2020 (Fig. 1). In addition to forecasting the overall annual NSCLC patient counts, the model was designed to allow stratification of these estimates across LOTs (second and third line) and histology (squamous and non-squamous).

To estimate the annual number of new advanced NSCLC cases in each country, age- and sex-stratified incidence rates were applied to United Nations (UN) world population data. Historical incidence rate data from the year 2000 through the current year of available data were used to construct a segmented linear regression model to determine 1) average annual incidence rate estimates; and 2) annual percent change (APC) estimates for the incidence rates [8]. The APC incidence estimates were then used to project future annual NSCLC incidence rates. Analyses to determine APC estimates were conducted using Joinpoint software [9].

Proportions of patients by histology and stage at diagnosis were then applied to the annual lung cancer incidence counts for each country. Patients classified as having SCLC were excluded from future counts in the model. NSCLC incidence counts were then stratified according to the proportion of patients with early- versus advanced-stage disease at diagnosis (early stage = stages I, II, and IIIA; advanced stage = stages IIIB and IV). Data from published materials describing NSCLC progression from early to advanced stage over a 10-year time interval were modeled using a Weibull distribution. The value for the α scale parameter was calculated using five-year recurrence data, and the value for the β shape parameter (i.e., accelerating or decelerating failure) was fit to the published survival curves using the least squares method. In this model, patients without recurrence were assumed to be cured of disease. Patients who receive non-systemic best supportive care (BSC) and do not receive systemic treatment from a medical oncologist were excluded from the total advanced NSCLC patient count. The proportion of those receiving systemic treatment by LOT and tumor histology (squamous or non-squamous) was applied to the annual case counts to calculate the annual country-specific patient counts by LOT. Estimated annual mortality was modeled in the same method as the recurrence calculation.

### Model parameterization

Three types of data sources were used to parameterize the PCM. First, publicly available internet-based datasets, analysis tools, and reports of regional or national cancer registries were searched to obtain lung cancer incidence, prevalence, and mortality data (age- and gender-stratified); the proportion of patients by stage of NSCLC at diagnosis (stages I to IV); and morphology (NSCLC versus SCLC). Table 1 shows the data sources and types of data elements extracted from registry sources for each country.

**Table 1:** Publicly available internet-based cancer registry data sources and data elements available for extraction by country

Country	Data sources	Data available	Incidence estimates data years^a^
France	EUREG, InVS [29, 30]	Incidence and mortality	2000–2009, 2012
Germany	Robert Koch Institute [31]	Incidence and mortality	2000–2012
Italy	AIRTUM/ITACAN [32, 33]	Incidence and mortality	2000–2009
Spain	EUREG [29]	Incidence and mortality	2000–2007

Key: *InVS* Institut de Veille Sanitaire, *KRN* Krajowy Reejestr Nowotworow

^a^Years refer to availability of incidence data; for modeling purposes, data were extracted only back through the year 2000

The second data source for parameterizing the PCM included a comprehensive literature review on NSCLC epidemiology and treatment patterns. The comprehensive literature review used an approved set of database search terms, review of all retrieved titles/abstracts and relevant full-text papers, and quality assessment of the identified studies. The literature review of MEDLINE and EMBASE (2005–January 2015) identified 163 studies for full-text review, and 109 studies contained relevant data for extraction (see Additional file 1). As part of the quality control process for study screening, the literature review protocol required that a second independent reviewer spot-check 10% of the retained articles and 10% of the articles excluded during screening to ensure that the articles were being screened according to pre-specified inclusion and exclusion criteria. Any disagreement over inclusion was resolved by discussion with a third, independent reviewer. Data were extracted by a single reviewer and were independently verified and validated by a second reviewer. When multiple data sources and parameter estimates were available, the most robust country-specific data source was selected as the default PCM parameter estimate. Quality was assessed by two independent reviewers by scoring studies across four parameters: 1) sample representativeness (selection bias); 2) study design; 3) quality of reporting (detection bias); and 4) attrition bias (see Additional file 2). If no country-specific data were available, aggregate estimates from other European countries were used to parameterize the PCM. Survival estimates for relapsed and refractory advanced NSCLC came from recent multinational randomized controlled studies [10, 11]. Finally, data from a retrospective chart review study of NSCLC treatment patterns in Europe were used to estimate the proportion of patients receiving second and third LOT by squamous or non-squamous tumor histology [12, 13].

APC incidence for each age and sex cohort was calculated, and the modeled estimates were used as the baseline incidence rate counts per year. Table 2 shows the incident APC estimates for the most recent Joinpoint segment.

**Table 2:** Annual percent change estimates by country, gender, and age for the last Joinpoint segment of the model

Country and sex		Age cohort									
		40–44	45–49	50–54	55–59	60–64	65–69	70–74	75–79	80–84	85+
France	M	−4.8^a^	-4.1^a^	−0.8	0.8	1.4^a^	1.5^a^	0.6	0.6	0.7	4.1
	F	−1.8	1.2	8.2^a^	8.6^a^	8.5^a^	7.9^a^	6.0^a^	4.1^a^	5.6	9.3^a^
Germany	M	−6.4	−5.1	−6.8	−1.3	−0.4	−0.6	−2.7	−4.8	−0.6	− 0.1
	F	−2.8	−2.7	−1.9	2.6	6.4	6.1	2.1	−2.2	1.9	3.2
Italy	M	−7.3^a^	−5.0^a^	−4.6^a^	−3.5^a^	−0.9	−2.4^a^	−3.2^a^	−1.4^a^	− 3.8	2.2^a^
	F	−1.9	3.7^a^	7.6^a^	8.2^a^	3.1^a^	2.4^a^	1.2	1.9^a^	3.8^a^	6.4^a^
Spain	M	−4.5^a^	−2.3	−2.0	1.9	−0.1	0.8	−0.5	−0.2	−1.5	−7.6
	F	1.9	9.5	14.9^a^	4.5	10.2^a^	4.3	7.2^a^	−1.5	−1.0	−1.8

^a^Indicates that the annual percent change estimate is significant at *P* < 0.05

Country-specific estimates for the morphology and histology of lung cancer are presented in Table 3. The country-specific distributions of NSCLC stage at diagnosis were derived from a large observational, prospective cohort study on disease characteristics, treatment patterns, and outcomes related to treatment in select European countries (EPICLIN). Country-specific estimates of the second and third LOT treatment rate for squamous and non-squamous advanced NSCLC came from chart review studies of patients receiving care from a medical oncologist/pulmonologist. To validate model results, patient count forecasts were compared to published estimates of 2012 worldwide cancer incidence and mortality estimates by WHO and against published population-based cancer reports [1, 14, 15].

**Table 3:** Model parameter estimates and data sources

Parameter		France	Germany	Italy	Spain	Model code color
Proportion non-small cell cases at diagnosis (%)		83.1	81.2	84.8	86.6	Orange
	Source	[34]	[35]	[32]	[36]	
Squamous (%)		38.3	35.9	32.2	40.5	
Non-squamous (%)		61.7	64.1	67.8	59.5	
	Source	[12, 13]	[12, 13]	[32]	[12, 13]	
Stage at diagnosis (%)	I	7.5	13.7	10.0	7.4	Purple
	II	7.9	8.3	5.9	6.2	
	III	25.2	33.4	23.3	33.4	
Proportion of stage III patients who are IIIb (%)		49.6	60.8	63.1	68.3	
	IV	52.9	41.3	53.5	48.5	
	Unknown	6.5	3.3	7.3	4.5	
	Source	[37]				
Five-year rate of distant recurrence of the tumor from stage (%)	I	25.8				Blue
	II	45.8				
	IIIa	44.8				
	Source	[38]				
Median OS (months)	Squamous NSCLC	6.0				
	Source	[10]				
	Non-squamous NSCLC	9.4				
	Source	[11]				
Proportion of advanced NSCLC patients referred to BSC (%)		11.7%				Aqua blue
Proportion eligible for systemic treatment (%)		88.3%				
	Source	[36]				
Proportion of squamous patients receiving (%)	First LOT	77.9	89.2	90.0	97.5	Green
	Second LOT	28.6	37.8	42.2	39.2	
	Third LOT	10.4	9.5	10.0	2.5	
Proportion of non-squamous patients receiving (%)	First LOT	89.5	93.8	92.3	99.1	
	Second LOT	46.8	52.3	57.7	54.3	
	Third LOT	12.9	18.5	8.7	16.4	
	Source	[13]				

### NSCLC PCM sensitivity analysis

A probabilistic sensitivity analysis (PSA) was conducted in which key model inputs, including APC, morphology, stage at diagnosis, tumor histology, five-year recurrence rate, proportion of patients receiving each LOT, and overall survival (OS) rate, were parameterized using appropriate distributions and standard errors calculated from the 95% confidence intervals (CIs). Proportions were parameterized using beta distributions, APC incidence estimates were parameterized using normal distributions, time-to-event data were parameterized using Weibull distributions, and stage at diagnosis was parameterized using a Dirichlet distribution. A total of 1000 simulations were conducted, from which the mean, 95% low CI, and 95% high CI estimates were then calculated to obtain the probabilistic patient counts.

## Results

**Fig. 2:**
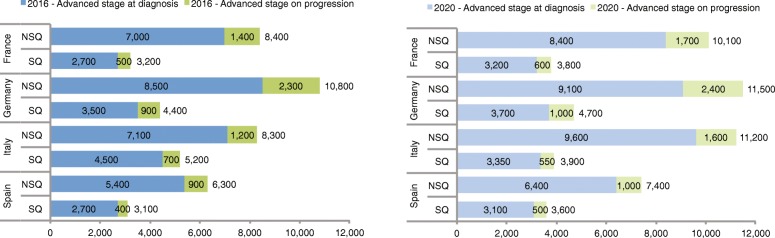
Model projections of the number of advanced-stage NSCLC patients receiving second-line therapy in 2016 and 2020 by histology and country. Key: NSQ – non-squamous; SQ – squamous

**Fig. 3:**
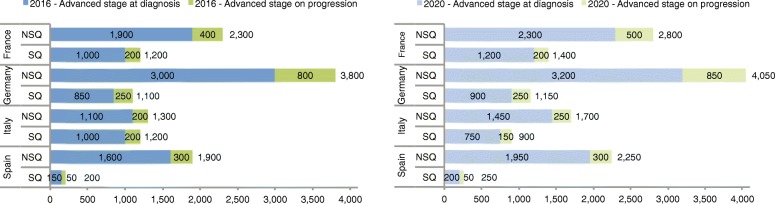
Model projections of the number of advanced NSCLC patients receiving third-line therapy in 2016 and 2020 by histology and country. Key: NSQ – non-squamous; SQ – squamous

The PCM forecasts an increase in the previously treated advanced NSCLC population for all countries between 2016 and 2020 (Table 4, Figs 2 and 3). The largest growth rate of second LOT-eligible patients was observed in France, where the model forecasts a compound annual growth rate of over 4%.

**Table 4:** Deterministic and probabilistic model projections of the number of advanced NSCLC patients receiving second- and third-line therapy in 2016 and in 2020 by country

Country	Burden of illness measure	Year of estimate							
		2016				2020			
		Deterministic	Probabilistic			Deterministic	Probabilistic		
		Mean	Mean	Lower 95% CI	Upper 95% CI	Mean	Mean	Lower 95% CI	Upper 95% CI
France	Second LOT	11,600	11,700	10,500	12,800	13,900	13,900	12,500	15,400
	Third LOT	3500	3500	3200	3800	4200	4200	3800	4500
Germany	Second LOT	15,100	15,200	13,600	16,700	16,200	16,200	14,600	17,900
	Third LOT	4900	4900	4500	5400	5200	5300	4800	5700
Italy	Second LOT	13,500	13,600	11,900	15,600	15,100	15,600	13,500	18,200
	Third LOT	2500	2500	2200	2800	2600	2700	2400	3100
Spain	Second LOT	9400	9600	8400	11,100	11,000	11,800	9600	14,700
	Third LOT	2100	2200	1900	2400	2500	2600	2200	3300

Key: *CI* confidence interval, *LOT* line of therapy, *NSCLC* non-small cell lung cancer

## Discussion

Lung cancer is the leading cause of cancer-related death in Europe and often remains undiagnosed until the tumor has reached an advanced stage, where patient survival is poor. New immune-oncology therapies approved for previously treated patients have significantly improved survival in clinical study and have the potential to improve the duration and quality of life for patients. However, data on the size of the treatment-refractory population in Europe that is eligible for these new therapies are limited. Reliable estimates of the population likely to receive treatment for refractory advanced NSCLC help improve the understanding of the potential clinical and economic impact of these new treatments.

In this study, we developed an Excel-based PCM to project country-level estimates of the number of patients receiving treatment for relapsed and refractory advanced NSCLC in France, Germany, Italy, and Spain over the five-year period from 2016 to 2020. For all countries, the model forecasts an overall increase in the number of patients with advanced-stage squamous and non-squamous NSCLC expected to receive second- and third-line treatment in the year 2020. The increase in patient numbers was driven primarily by growth in the incidence of NSCLC among women. Across the majority of countries and age groups, lung cancer incidence rates for females continued to increase, most notably in France. These data are in line with the current understanding of tobacco-smoking patterns and the epidemiology of lung cancer. Tobacco smoking is the leading risk factor for lung cancer and has been strongly associated with lung cancer, particularly with squamous SCLC and NSCLC [16]. In France, tobacco smoking has been attributed to > 80% of lung cancer deaths [17]. Reflecting the earlier decline in smoking prevalence among men, lung cancer rates in men have either declined or plateaued, while rates of lung cancer in women continued to rise or, only recently, modestly decreased [18–20].

Lung cancer screening plays an important role in survival and disease trends for advanced NSCLC patients. The National Lung Screening Trial (NLST) found early screening of high-risk individuals with low-dose computed tomography (LDCT) reduces lung cancer-related mortality by 20% [21]. Following publication of these results, some professional organizations have recommended early screening in high-risk populations, but adoption of early screening efforts has been hindered by concerns related to radiation exposure, false positives, and cost [22]. Based on results of the NLST, ESMO clinical practice guidelines report LDCT reduces lung cancer-related mortality, but the guidelines cite many reasons to not recommend large-scale implementation, notably cost-effectiveness concerns [23]. Lung cancer screening rates were not included in the described PCM, but significant changes to screening rates could affect the number of patients eligible for treatment in the future and the validity of the model estimates.

There are limited published data on the current and future burden of relapsed and refractory advanced NSCLC in Europe; the patient count forecasts were validated against WHO data and published lung cancer reports. Forecasted results aligned with these published estimates and country trends in smoking and new lung cancer incidence. As an example, the model forecasts a compound annual growth rate of 4.5% in the number of patients receiving treatment in France, which matched the modeled growth rate published in a 2015 report by the French Institute for Public Health Surveillance (InVS) [14]. The InVS report projected 2015 lung cancer incidences of 45,222, which is 0.5% lower than the 45,456 forecasted by the PCM. Using simple linear regressions of historical lung cancer incidence data by age and gender cohorts, combined with clinical treatment pattern data, has produced accurate patient forecasts.

The limitations of this study are mainly related to data used to parameterize the PCM and assumptions made in the PCM structure. The PCM assumes 11.7% of patients receive palliative care only and 88.3% are eligible to receive systemic chemotherapy under the care of a medical oncologist. Due to the scarcity of published data on treatment rates of advanced NSCLC patients in France, Germany, Italy, and Spain, treatment rates utilized in the PCM were derived from a single retrospective chart review study of patients receiving first, second, and third LOT in the respective countries. However, published treatment rates reported for Canada, the United States, and the United Kingdom were generally lower (range: 26 to 58%) than the rates used in the PCM, which may indicate that the PCM currently overestimates the treated population [24–28]. The PSA conducted as part of the modeling exercise provides a range of higher and lower patient number projections as the treatment rate is modified. Recurrence data were from population-based estimates reported in a recent study from Italy. While this study provides a robust estimate of the rates of recurrence, it is unknown to what degree different patient populations (in terms of country) or other factors, such as tumor histology, may influence this rate. Additionally, this study reviewed patient data from 2002 to 2005, which may differ from more current, real-world recurrence rates. Due to data limitations, we assumed that the median OS was the same across all countries, although different between squamous and non-squamous histology patients. Default OS estimates for all squamous and non-squamous advanced NSCLC patients were from the docetaxel arm of recent pivotal trials for nivolumab and may underestimate real-world survival rates as new treatments are adopted. However, the impact of varying this parameter did not significantly affect patient count estimates. Finally, we assumed that the APC estimate was a robust estimator of the trend in lung cancer incidence rates. All trend estimates, regardless of statistical significance, were applied to the last modeled estimated incidence rate for the data.

## Conclusions

A modeling exercise was completed to estimate the future number of patients with advanced, refractory NSCLC eligible for systemic treatment in France, Germany, Italy, and Spain. The model forecasts an overall increase in the number of patients with advanced-stage squamous and non-squamous NSCLC likely to receive second- and third-line treatment in the year 2020 for all countries. Trends in advanced NSCLC treatment appear to be strongly gender-dependent. The increase in relapsed and refractory advanced NSCLC patients receiving treatment in 2020 is driven by growth in the incidence of lung cancer among women. Given the high mortality rates associated with advanced disease, the results of the model highlight the need for effective treatment options that can improve survival and for robust surveillance data to accurately quantify the burden of disease.

## Additional files

Additional file 1: Comprehensive literature search results. Diagram summarizing results of the comprehensive literature search. (PDF 218 kb)

Additional file 2: Literature quality assessment criteria. Scoring criteria for the assessment of literature quality. (PDF 85 kb)

## Abbreviations

APC: Annual percent change
BSC: Best supportive care
CI: Confidence interval
InVS: Institut de Veille Sanitaire
KRN: Krajowy Reejestr Nowotworow
LDCT: Low-dose computed tomography
LOT: Line of therapy
NLST: National Lung Screening Trial
NSCLC: Non-small cell lung cancer
NSQ: Non-squamous
OS: Overall survival
PCM: Patient count model
PSA: Probabilistic sensitivity analysis
SCLC: Small cell lung cancer
SQ: Squamous
UN: United Nations
WHO: World Health Organization
