# Supplementary figures and images for: The present and future burden of previously treated advanced non-small cell lung cancer (NSCLC) by histology and line of therapy in France, Germany, Italy, and Spain: model-based predictions
Source: Popul Health Metr. 2018 Nov 26;16:17. doi: 10.1186/s12963-018-0174-4 (PMC6257974; doi:10.1186/s12963-018-0174-4)

## Additional file 1. Comprehensive Literature Search Results

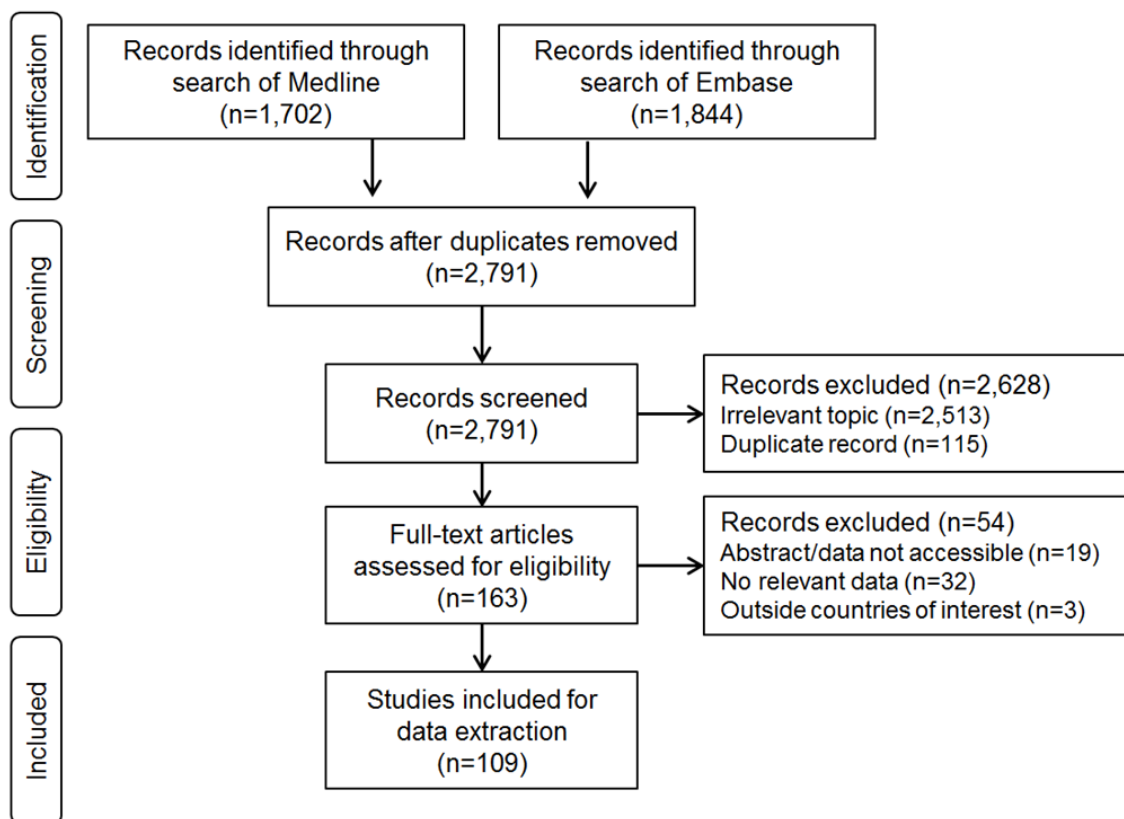

Supplement: Supplementary file 1 — Comprehensive literature search results. Diagram summarizing results of the comprehensive literature search. (PDF 218 kb) [file 12963_2018_174_MOESM1_ESM.pdf]
