# Supplementary material for: The present and future burden of previously treated advanced non-small cell lung cancer (NSCLC) by histology and line of therapy in France, Germany, Italy, and Spain: model-based predictions
Source: Popul Health Metr. 2018 Nov 26;16:17. doi: 10.1186/s12963-018-0174-4 (PMC6257974; doi:10.1186/s12963-018-0174-4)
Supplement: Supplementary file 2 — Literature quality assessment criteria. Scoring criteria for the assessment of literature quality. (PDF 85 kb) [file 12963_2018_174_MOESM2_ESM.pdf]

Additional file 2: Literature Quality Assessment Criteria

Quality Parameter	Score		
	2	1	0
**Sample representativeness (selection bias)**	Multi-site tumor database, consecutive patients, multiple sites/countries/regions	1 practice or hospital site and consecutive patients, convenience sample	Not described, none of the above
**Study design**	Prospective, longitudinal (cross-sectional for prevalence is also appropriate)	Cross-sectional, case control, retrospective evaluation of patient and/or tumor characteristics	Not described, none of the above
**Quality of reporting (detection bias)**	Detailed description of patient selection and outcome definitions following established standards	Details on patient selection and outcome definitions are available but do not follow established standards	Details on how patients were selected and outcomes were missing or limited (eg, diagnostic criteria for advanced stage disease not provided)
**Attrition bias**	Loss to follow-up is minimal and the majority of eligible/enrolled patients have information on outcomes of interest	Loss to follow-up is described and is not either major or minor	Loss to follow-up is either not described for applicable outcomes or a large portion of eligible/enrolled patients are missing information for outcomes of interest
